# Risk factors for bronchiolitis severity: A retrospective review of patients admitted to the university hospital from central region of Slovenia

**DOI:** 10.1111/irv.12587

**Published:** 2018-08-09

**Authors:** Ajda Praznik, Neža Vinšek, Ana Prodan, Vanja Erčulj, Marko Pokorn, Tatjana Mrvič, Darja Paro, Uroš Krivec, Franc Strle, Miroslav Petrovec, Marta Žnidaršič Eržen, Štefan Grosek

**Affiliations:** ^1^ Faculty of Medicine University of Ljubljana Ljubljana Slovenia; ^2^ RhoSigma Ljubljana Slovenia; ^3^ Department of Infectious Diseases University Medical Centre Ljubljana Ljubljana Slovenia; ^4^ Chair of Infectious Diseases Faculty of Medicine University of Ljubljana Ljubljana Slovenia; ^5^ Department of Neonatology University Children's Hospital University Medical Centre Ljubljana Slovenia; ^6^ Chair of Pediatrics Faculty of Medicine University of Ljubljana Ljubljana Slovenia; ^7^ Pulmonology Department University Children's Hospital University Medical Centre Ljubljana Slovenia; ^8^ Chair of Microbiology and Immunology Faculty of Medicine University of Ljubljana Ljubljana Slovenia; ^9^ Institute of Microbiology and Immunology Faculty of Medicine University of Ljubljana Ljubljana Slovenia; ^10^ Pediatric Clinical Department Community Health Centre Ljubljana Ljubljana Slovenia; ^11^ Department of Pediatric Surgery and Intensive Care University Medical Centre Ljubljana Ljubljana Slovenia

**Keywords:** bronchiolitis, respiratory viruses, risk factors, RSV

## Abstract

**Aim:**

Study's objective was to identify risk factors associated with bronchiolitis severity.

**Methods:**

A retrospective chart review of all children <2 years old diagnosed with bronchiolitis at the University Medical Centre Ljubljana between May 2014 and April 2015, who were treated as outpatients (paediatric emergency department, PED group) or as inpatients in the standard hospital setting (WARD group) or in the paediatric intensive care unit (PICU group). Detection of respiratory viruses in nasopharyngeal swab was accomplished by RT‐PCR. Severity was assessed by Wang Respiratory Score and hospitalization longer than 24 hours.

**Results:**

The study included 761 children. The three most frequently detected viruses were respiratory syncytial virus (RSV), human rhinovirus (hRV) and human bocavirus (hBoV) (57.5%, 272/473; 25.6%, 121/473; 18.4%, 87/473). Patient groups differed in Wang Respiratory Score for the severity of bronchiolitis (*P* < 0.001). No differences regarding the causative viruses were found. There was a lower proportion of children with the presence of more than one virus in PICU group compared to other two groups (*P* = 0.017). The three groups significantly differed in age, birthweight, comorbidities, bronchodilator treatment and antibiotic usage. However, multiple regression analysis revealed that younger age and the use of antibiotics were associated with bronchiolitis severity defined as hospitalization for >24 hours.

**Conclusions:**

Respiratory syncytial virus, hRV and hBoV were the most frequently detected viruses. The majority of patients admitted to the PICU had only one virus detected. Younger age and the use of antibiotics were associated with bronchiolitis severity.

## 1 INTRODUCTION

Bronchiolitis is a potentially life‐threatening viral respiratory infection that affects children under 2 years of age.^1^ It is common and occurs worldwide. The bronchiolitis mortality rate is approximately 2 per 100 000 infants and is higher in developing than in developed countries.^2^ The most commonly identified causative agent is respiratory syncytial virus (RSV).^2^ Other viruses implicated in the aetiology of bronchiolitis include human metapneumovirus (hMPV), parainfluenza virus (PIV), influenza virus A and B virus (InfV), human rhinovirus (HRV), human coronavirus (HCoV), adenovirus (AdV), enterovirus (EV) and human bocavirus (HBoV).^3^

The majority of children with bronchiolitis can be managed as outpatients; approximately 2%‐3% of patients younger than 1 year are hospitalized, usually during the seasonal RSV epidemics that occur in cold months.^1^

Treatment of bronchiolitis is symptomatic and focuses on the maintenance of hydration, oxygenation and antipyresis. In severe cases, intensive care management may be needed with continuous positive airway pressure (CPAP), intubation and mechanical ventilation. Some infants with secondary bacterial infection need antibiotic treatment.^4^, ^5^, ^6^ In infants with pre‐existing medical conditions and immunodeficiency, complications such as acute respiratory distress syndrome (ARDS), bronchiolitis obliterans, congestive heart failure and secondary bacterial infection may develop.^4^, ^5^, ^6^, ^7^, ^8^ Although there is no universally accepted measure for the assessment of bronchiolitis severity, the Wang Respiratory Score, using clinical observation (general appearance, respiratory rate, presence of wheezing and retractions), is widely used.^9^ Children with bronchiolitis and Wang Respiratory Score 3 or less can be managed on an outpatient basis, while children with scores 4‐8 usually require hospitalization and those with even higher scores are admitted to the PICU.^10^

The objective of our study was to ascertain demographic characteristics, clinical findings and presumptive aetiologic agents (respiratory viruses demonstrated in nasopharyngeal swab) associated with bronchiolitis severity defined as length of hospitalization for >24 hours.

## 2 METHODS

### 2.1 Ethics

The study was approved by the National Medical Ethics Committee of the Republic of Slovenia (KME 37/09/15) (NMEC RS).

### 2.2 Patient selection

The study was conducted at the University Medical Centre Ljubljana (UMCL), a university hospital serving the central region of Slovenia with a population of around 700 000 inhabitants (approximately one‐third of all Slovenian population). We performed a retrospective chart review of all patients with bronchiolitis <2 years of age, referred to the three paediatric departments, that is UMCL Children's Hospital, Department of Infectious Diseases and Paediatric Intensive Care Unit (PICU) between 1 May 2014 and 30 April 2015. We excluded patients that had been previously diagnosed with asthma, and all previous episodes if a patient had more than one episode of bronchiolitis in studied time span. Patients were identified through a query of the electronic medical record based on a bronchiolitis diagnosis (ICD‐10 code J21.0‐9).

### 2.3 Study design/data collection

Electronic medical records of patients included in the study were reviewed, and statistical analysis was performed for the following clinical and laboratory data: gender, chronological age at admission, prematurity (defined as birth before 37 weeks of gestation), birthweight, history of allergies, number of previous bronchiolitis episodes, clinical manifestations of bronchiolitis using the Wang Respiratory Score,^9^ comorbidities (chronic lung disease, congenital heart disease, immune deficiency or neuromuscular diseases), body temperature at admission, treatment with bronchodilators, antibiotics or supplemental oxygen and respiratory virus detected in the nasopharyngeal swab. Only the first swab taken from individual patient was included. Swabs were routinely taken from patients in two departments, while in another one sampling was sparse. High season and low season of incidence were also included as variables. The seasons were previously determined with cluster analysis of the demographic structure of children diagnosed with bronchiolitis in the period from 2010 to 2013 as reported by the National Institute of Public Health (NIPH). According to the data from the NIPH, in 2014/2015, the respiratory syncytial virus (RSV) seasonal epidemic in the Republic of Slovenia started in November, peaked in December and ended in February.^11^ Viruses were detected in nasopharyngeal swabs using reverse transcriptase polymerase chain reaction (RT‐PCR), and all samples were tested for the presence of RSV, hMPV, HBoV, HRV, PIV, InfV (A and B), HCoV, EV and AdV.^12^ Using rRT‐PCR, the sensitivity of the nasopharyngeal swab is above 90% compared to a composite gold standard.^13^, ^14^ For 288 patients, no swab analysis was performed.

Bronchiolitis severity was assessed by Wang Respiratory Score and as a duration of hospitalization for >24 hours.

### 2.4 Statistical analysis

Numerical data were presented as median (range) and categorical as frequencies (percentages). The differences in categorical variables among the three groups were tested using chi‐square test or likelihood ratio test; Kruskal‐Wallis test was performed for numerical variables.

The clinical and laboratory findings were compared between the three groups of patients, defined according to the site of management: patients treated as outpatients (paediatric emergency department, PED group), patients treated in the standard hospital setting (WARD group) and children admitted to the paediatric intensive care unit (PICU group). Multiple logistic regression analysis was used to identify key variables associated with severe bronchiolitis, defined with the duration of hospitalization for >24 hours.

A *P‐value *< 0.05 was considered statistically significant. All analyses were performed using SPSS 23.0.

## 3 RESULTS

The study group comprised 761 children with bronchiolitis. There was a male predominance with 468 (61.5%) boys. Of 761 children, 138 were treated as outpatients (PED group), 599 were hospitalized at a regular paediatric unit (WARD group), and 24 were treated in a paediatric intensive care unit (PICU group). Nasopharyngeal swabs were taken in 473/761 (62%) children.

**Figure 1:**
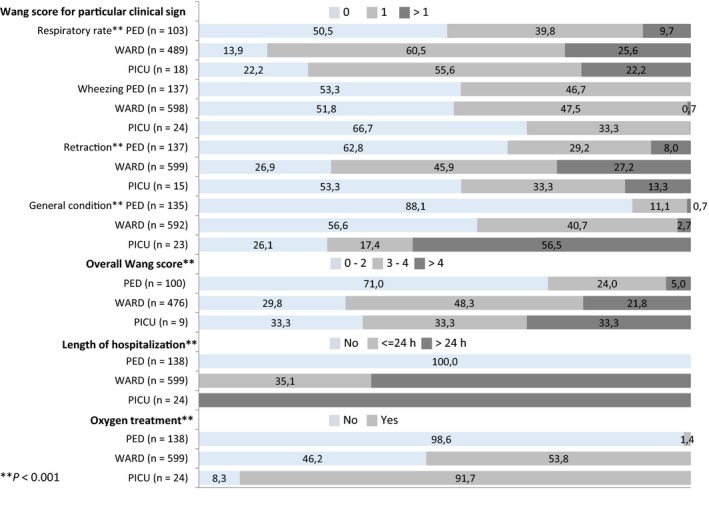
Association between Wang score for particular clinical sign and Wang score and type of management

Of 623 patients admitted to hospital, 413 (54%) were hospitalized for >24 hours. The median length of hospital stay was 12 days in PICU group and 2 days in WARD group (*P* < 0.001). The three study groups differed in Wang's score for particular clinical sign parameter (*P* < 0.001) apart from wheezing, and in the need for supplemental oxygen treatment (*P* < 0.001)—only a few patients required oxygen treatment in the outpatient PED group, while almost all patients needed oxygen treatment in PICU group. The three study groups differed in Wang score (*P* < 0.001) (Figure 1).

They also differed in several clinical and microbiological characteristics (Table 1). The mean age was 13.5 months in outpatient (PED) group, 10 months in inpatient (WARD) group and 1.5 months in the PICU group (*P* < 0.001). The proportion of premature children was the highest in the PICU group, lower in the WARD group and the lowest in the PED group. The proportion of children with comorbidities was statistically significantly higher in PICU group than in PED or WARD group (*P* < 0.001). Comparison of children receiving antibiotic treatment among the three groups showed significant differences as the majority of children in PICU group received antibiotics compared to a much lower proportion in the WARD and PED group.

**Table 1:** Association between demographic, clinical and microbiological characteristics of patients and type of management

	PED No = 138		Ward No = 599		PICU No = 24		*P*‐value
	n	%	n	%	N	%	
Gender							
Female	57	41.3	225	37.6	11	45.8	0.542^a^
Chronological age							
Median (range)	13.5 (0‐23)		10 (0‐23)		1.5 (0‐14)		<0.001^b^
Prematurity	9/98	9.2	89/463	19.2	9/21	42.9	0.001^a^
Caesarean section	20/87	23	98/396	24.7	5/14	35.7	0.592 b
Allergies	10/126	7.9	38/554	6.9	1/23	4.3	0.804^b^
Birthweight (g)							
Median (range)	3.500 (935‐4.600)		3.290 (610‐5.520)		3.020 (900‐3.800)		0.006^b^
High season	96	69.6	390	65.1	20	83.3	0.125^a^
>1 episode of bronchiolitis	12	8.7	84	14.0	0	0	0.039^a^
Comorbidities	3	2.2	20	3.3	8	33.3	<0.001^a^
Body temperature at admission							
<37.1°C	84/133	63.2	321/572	56.1	9/16	56.3	0.202^c^
37.1‐37.8°C	26/133	19.5	159/572	27.8	6/16	37.5	
>37.8°C	23/133	17.3	92/572	16.1	1/16	6.3	
Bronchodilators	66	47.8	378	63.3	16	66.7	0.003
Antibiotics	5	3.6	94	15.7	23	95.8	<0.001^a^
Number of viruses detected in individual swab							
1	37/62	59.7	263/387	68.0	22	91.7	0.073^c^
2	20/62	32.3	93/387	24.0	1	4.2	
3	4/62	6.5	29/387	7.5	1	4.2	
4	1/62	1.6	2/387	0.5	0	0	
RSV	34/62	54.8	223/387	57.6	15/24	62.5	0.808^b^
Rhinovirus	15/62	24.2	100/387	25.8	6/24	25	0.961^b^
Bocavirus	13/62	21	70/387	18.1	4/24	16.7	0.841^b^

Prematurity: birth before 37 wk of gestation. Season: High from December to April.

PED, Paediatric Emergency Department; PICU, Paediatric Intensive Care Unit; RSV, Respiratory Syncytial Virus.

Values are shown as frequencies (percentages) for categorical and as median (range) for numerical variables.

a chi‐square test.

b Kruskal‐Wallis test.

c Likelihood ratio test.

The most common viruses present in nasopharyngeal swab were RSV (272/473, 57.5%), HRV (121/473, 25.6%) and HBoV (87/473, 18.4%). The proportions of patients with individual virus were similar in children who were treated as outpatients, in those who were treated in the standard hospital setting and in the ICU group. Analysis of the presence of more than one virus in nasopharyngeal swab revealed that RSV and hBoV were the two most frequently simultaneously detected viruses and that the proportion of patients with more than one of detected viruses in individual swab was statistically significantly lower in PICU than in PED or WARD (*P* = 0.017) (Table 1).

Multiple logistic regression analysis showed that out of several factors only younger chronological age (*P* < 0.001) and treatment with antibiotics (*P* = 0.003) were associated with severe bronchiolitis defined as hospitalization >24 hours. (Table 2).

**Table 2:** Variables associated with severe bronchiolitis, defined as hospitalization for >24 h, using multiple logistic regression

Variables	OR (95% CI)	*P*‐value
Male gender	1.07 (0.44; 2.58)	0.884
Chronological age (mo)	0.87 (0.81; 0.93)	**<0.001**
Maturity	0.69 (0.14; 3.38)	0.646
Caesarean section	1 (0.36; 2.79)	0.996
Birthweight (g)	1 (1; 1)	0.786
Allergies	1.92 (0.23; 16.16)	0.547
More than one episode	2.62 (0.6; 11.42)	0.201
High season	1.31 (0.51; 3.38)	0.572
CRP (g/L)	1.01 (0.99; 1.03)	0.474
Leucocytes (10^9/L)	1 (0.92; 1.09)	0.965
Bronchodilators	2.06 (0.8; 5.34)	0.136
Antibiotics	13.18 (2.43; 71.64)	**0.003**
Body temperature	‐	0.184
>37.8°C	1.81 (0.68; 4.81)	0.234
37.1‐37.8°C	0.56 (0.18; 1.75)	0.317
<37.1°C	Ref.	‐
Comorbidities	0.13 (0.01; 1.64)	0.115
≥2 viruses detected	2.38 (0.87; 6.5)	0.091

OR, odds ratio; CI, confidence interval.

## 4 DISCUSSION

In the present study, we compared characteristics of children with bronchiolitis treated in the outpatient clinic, admitted to the ward or admitted to the paediatric intensive care unit. Age, prematurity, comorbidity and treatment with bronchodilators and antibiotics, differed in the three groups suggesting these factors relate to disease severity. The type of virus did not correlate with treatment group (Table 1). Further analyses revealed that chronological younger age and treatment with antibiotics independently were associated with hospitalization longer than 24 hours (Table 2).

Chronological age is known as the most important predictor of severe bronchiolitis. Similar to study, several others have found a significant association between the age of less than 6 months and a higher risk of hospitalization and severe bronchiolitis.^15^, ^16^, ^17^, ^18^ However, Grimwood et al did not find a significant correlation between children aged less than 2 months and bronchiolitis severity in a multivariate analysis.^19^ Hospitalization rates that are attributable to RSV bronchiolitis are usually the highest between 30 and 90 days after birth.^20^ This age coincides with the declining concentration of transplacentally acquired maternal anti‐RSV immunoglobulin, which protects infants against disease.^20^, ^21^

Certain studies have shown that prematurity is independently associated with more severe bronchiolitis,^15^, ^16^, ^22^ while the others have not found significantly higher rates of hospitalization and severe bronchiolitis among premature compared to full‐term infants.^17^, ^20^, ^23^ In the present study, the proportion of preterm children was highest in the PICU group and lowest in the PED group, which suggested a more severe bronchiolitis. However, multiple logistic regression model did not show a correlation between prematurity and severe bronchiolitis, maybe because our study did not differentiate between the very preterm, early preterm and late preterm infants.

In the present study, antibiotic treatment was significantly associated with severe bronchiolitis, which is in accordance with some previous reports.^24^, ^25^ Although the design of our study did not enable analysis of the appropriateness of antibiotic therapy, the finding that almost all patients in the PICU group received antibiotic treatment compared to only 3.6% in the PED group might suggest that bacterial superinfections lead to a more severe course of bronchiolitis.

Another frequently mentioned risk factor for severe bronchiolitis is comorbidity.^23^, ^26^, ^27^, ^28^ In our study, one‐third of children hospitalized in PICU had at least one comorbidity, while in the PED group the corresponding proportion was significantly lower. However, again, in the multiple logistic regression analysis comorbidities did not correlate with the severe course of bronchiolitis (Table 2) as shown also in some other studies.^1^, ^16^ One should take into account that our study did not analyse the association of comorbidities with specific virus types as done in mentioned studies.^5^, ^15^, ^26^, ^29^ Another limitation is that we did not analyse specific comorbidities, but only as a group determined by previous studies.^24^, ^30^, ^31^ This is because there were only 31 children with comorbidity and we believe that dividing the comorbidity group into smaller groups would not give reliable information.

The present study showed that RSV, hRV and hBoV were the most frequently present viruses in nasopharyngeal swab of children with bronchiolitis. The predominance of RSV is in agreement with several previous reports.^3^, ^5^, ^32^ The second most common pathogen is usually hRV, followed by InfV or PIV 5, 19, 33 while in our study hBoV ranked the third. Some reports indicated that hBoV is rarely detected as a single agent in hospitalized children with bronchiolitis, leading to speculation that this virus is more likely to be an innocent bystander than a true pathogen.^5^ However, Uršič et al have recently reported the first fatal case of an extremely severe bronchiolitis caused by hBoV in an immunocompetent child.^16^

The absence of association between virus type and characteristics of patients is in agreement with the findings of several previous articles.^6^, ^17^, ^18^ Nevertheless, in some reports, differences in the severity of the disease caused by various types of viruses or viral co‐infections were established.^1^, ^34^, ^35^

According to our study, multiple viruses detected in the nasopharyngeal swab were moderately associated with a more severe course of the bronchiolitis, although not significant (*P* = 0.091). Other studies proved that children with viral coinfection were less likely to be admitted to intensive care unit than children with single virus infection.^21^, ^22^ Possible explanation is that as one or more viral respiratory pathogens can be detected in the upper respiratory tract of as many as 30% of asymptomatic young children,^36^, ^37^ it is likely that in several patients with bronchiolitis and more than one virus detected in nasopharyngeal swab, one of the viruses is responsible for the acute infection while the others are innocent bystanders, possibly as a result of prolonged shedding after an already resolved infection.

It is not unusual that a child has a history of more than one episode of bronchiolitis.^38^ Studies suggest that repeated infections by RSV can be in part due to variability of the virus strains.^39^ Acute bronchiolitis can cause (transitory) anatomical and histological changes in lower respiratory tract that may prone to a more severe new episode.^29^ However, in accordance with some previous reports 40 in our study, the number of previous episodes of bronchiolitis did not correlate with bronchiolitis severity.

Our study also showed that the proportion of children who received bronchodilators was statistically different in the PED, WARD and PICU group (Table 1). This is probably due to the common practice in several countries (such as United States, Switzerland and Belgium) that children exhibiting a moderately severe bronchiolitis are more often given bronchodilators than those with mild or severe disease.^41^, ^42^, ^43^ The data on the efficiency of bronchodilators in bronchiolitis are conflicting.^41^ Many studies agree that bronchodilators may improve clinical symptom scores, but they do not affect disease resolution, need for hospitalization, or length of stay.^5^, ^44^ Nonetheless, Kellner et al concluded that bronchodilators produce modest short‐term improvement in clinical features of mild or moderately severe bronchiolitis.^45^ However, despite the difference in proportion of children that received bronchodilators being statistically significant, our multiple regression analysis showed that treatment with bronchodilators was not associated with bronchiolitis severity.

Our study has several limitations. Firstly, the sample size of a PICU group is relatively small. With only 24 patients, more detailed analysis was sometimes not possible. We believe that our results are reliable, but bigger sample would provide additional insights and some pertinent variable investigated might have been significant. Its retrospective design has limited our ability to review additional information about other possible risk factors, for example Apgar score, smoking exposure, maternal age, presence of preschool‐aged siblings, siblings attending day care and patient attending day care.^16^, ^40^ Also, nasopharyngeal swabs were not taken in all patients due to the policy of one of the departments not to routinely sample all bronchiolitis patients. Viral diagnosis was done on 62% of children, which could be a limitation to result interpretation. In addition, children with performed viral diagnosis were younger and were treated in the high seasonal months. Comparison of the three groups regarding virus repartition should be taken with precaution as the sample of children treated in the standard hospital setting with viral data available seems to be biased. Data are not missing at random, but are available to higher extent for younger children and for children treated in the high season months.

In conclusion, our study revealed that RSV, hRV and hBoV were the most frequently detected viruses in children with bronchiolitis. Chronological younger age and the use of antibiotics were associated with severe bronchiolitis defined as hospitalization longer than 1 day. Further prospective studies are needed to assess the importance of various respiratory viruses and host factors in this common paediatric illness.

## CONFLICT OF INTEREST

The authors have no competing interests.
